# Dens Evaginatus in Proximal Surface of Mandibular Premolar: A Rare Presentation

**DOI:** 10.1155/2012/603583

**Published:** 2012-09-13

**Authors:** Shanthi Viswanathan, Vezhavendhan Nagaraj, Sanguida Adimoulame, Sathish Kumar, Gaurav Khemaria

**Affiliations:** ^1^Department of Oral Pathology, Indira Gandhi Institute of Dental Sciences, Pillayarkuppam, Puducherry, India; ^2^Department of Pedodontics and Preventive Dentistry, Indira Gandhi Institute of Dental Sciences, Pillayarkuppam, Puducherry, India; ^3^MR Ambedkar Dental College and Hospital, 1/36 Cline Road, Cooke Town, Bangalore 560 005, India

## Abstract

Dens evaginatus is a developmental anomaly that is characterized by occurrence of an extra cusp-like structure projecting from the crown portion of the tooth. Unusual extension of enamel has been found in posterior teeth as enamel pearl or as cervical enamel extensions from the cementoenamel junction or at the furcation areas. We hitherto report a case of extra enamel formation from the proximal surface of the crown in a mandibular premolar, a finding that has previously not been reported.

## 1. Introduction

Exophytic growth of dental tissues is a rare presentation among the various anomalies of dental morphology. Dens evaginatus presents as a protuberance from the involved surface of the tooth and consists of an outer layer of enamel, a core of dentin, and sometimes a slender extension of pulp tissue [1]. This developmental anomaly is usually characterized by the occurrence of an extra cusp, shaped as a tubercle projecting from the occlusal surface of the tooth and is known under various names such as tuberculated cusp, tuberculum dentis, crown tubercle, tuberculum coronae, accessory tubercle, Leong's premolar, evaginatus odontoma, and occlusal pearl [1, 2]. Tuberculated structure in the anterior teeth known as the talon cusp involves the lingual or the labial aspect of the incisors. Among the posterior teeth, the premolars are commonly affected followed by the molars involving primarily the occlusal surface [3]. Such elevations or tubercle-like structure in the proximal surface of any tooth is a rare finding; here we describe one such condition—a dens evaginatus on the proximal surface of the mandibular first premolar—with a brief discussion on its pathogenesis.

## 2. Case

**Figure 1:**
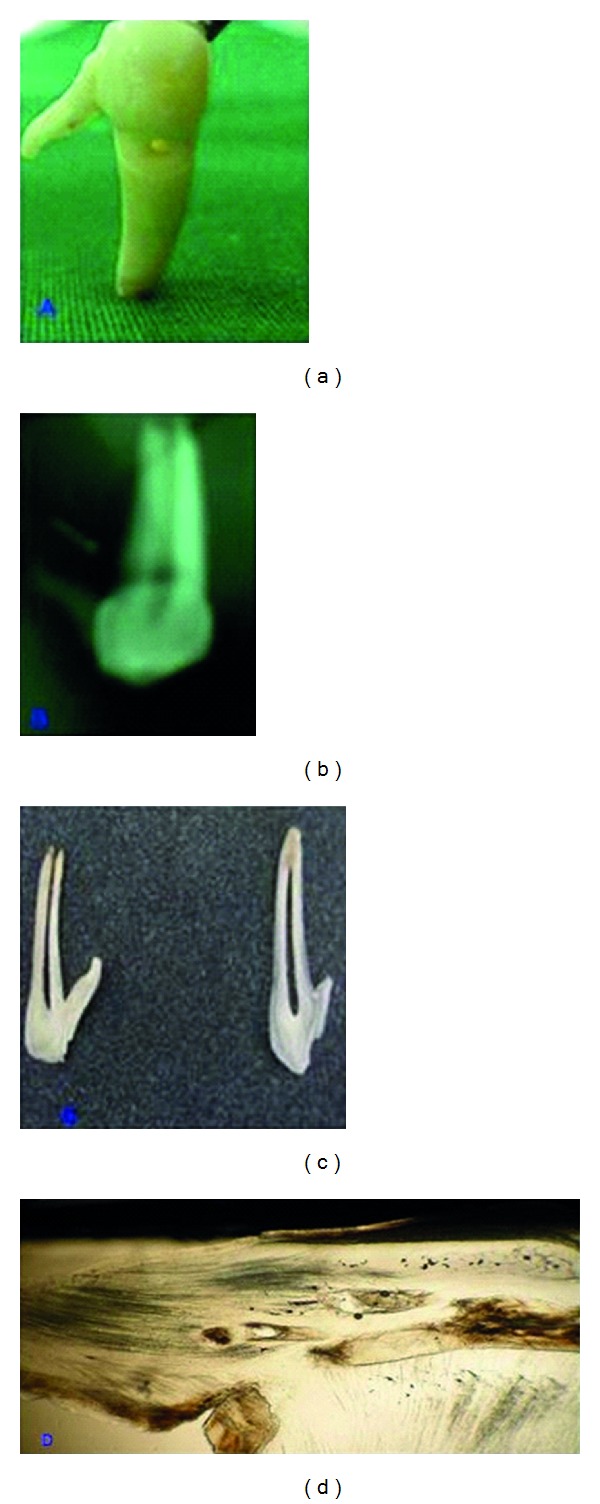
(a) Extracted tooth: showing presence of tubercle in relation to the mesial aspect of the mandibular premolar. (b) IOPA: showing presence of evagination with a radiopaque structure seen in mesial surface of tooth. (c) Tooth section: showing presence of tubercle attached to the mesial aspect of the mandibular premolar. (d) Ground section: showing evidence of enamel rods and dentinal tubules in the evaginated portion.

A 26-year-old man wanted to remove his right lower back tooth complaining of pain and mobility. On examination, cervical abrasion was present on the buccal aspect of the mandibular right first premolar with grade II mobility. The tooth was extracted as per patient's request. On examination, the tooth had a deep cervical abrasion on the buccal surface with pulpal exposure and presented with an open apex. An extension of the crown having the same color as that of enamel was seen arising from the distal aspect of the tooth just below the marginal ridge. It was triangular in outline with a base measuring around 0.5 cm along the smooth surface of the tooth with a pointed and a narrow apex (Figure 1(a)). Radiograph showed that the tubercle extending from the proximal surface of the crown had almost the same density as that of enamel without any difference of dentin or pulp (Figure 1(b)). Longitudinal section of the tooth was done (Figure 1(c)); ground section of the tooth revealed the tubercle in continuation with the proximal surface of the tooth showing enamel rods and dentinal tubules (Figure 1(d)). There was also evidence of interglobular dentin below the enamel surface along with foci of enamel-like structure entrapped within the dentinal tubules. There were also two brownish-black foci within the dentinal tubules associated with an empty space which could have been probably a calcified component. 

## 3. Discussion

The occurrence of dens evaginatus (DE) varies between one and four percent with a higher prevalence among people of Asian origin and is rare in white populations. It occurs in both primary and permanent dentitions, more frequently involving the mandibular premolars and rarely the molars, canines, incisors, and supernumerary teeth. It is five times more common in mandibular premolars. About 50% of cases have bilateral involvement of collateral teeth with a slight predilection for females [2].

DE projects above the adjacent tooth surface and results in formation of an accessory cusp whose morphology has been labeled as an abnormal tubercle, elevation, protuberance, evaginated odontoma, excrescence, and so forth [1, 3]. It's essential feature is an enamel covered tubercle that projects from the occlusal surface of an otherwise normal tooth.

A multifactorial etiology combining both genetics and environmental factors has been suggested for the formation of dens evaginatus [1, 3]. Mutations in the human EDA1, EDAR, and EDARADD genes often result in more severe phenotypes resulting in tooth loss and malformation [4]. During the bell stage of tooth formation, this anomaly is caused by internal enamel epithelium and the adjacent odontogenic mesenchyme evaginating into the stellate reticulum of the enamel organ [5]. Reports have shown that the enamel knots were found to act as central regulators of tooth development since they link cell differentiation to morphogenesis [3, 4, 6, 7]. Thesleff and Sharpe [7] have proposed that the enamel knot signals, together with mesenchymal signals, regulate the patterning of the cusps and hence the shape of the tooth crown. The primary enamel knot regulates the advancing cuspal morphogenesis of the crown through expression of molecules such as fibroblast growth factors (FGF-4, FGF-9), transforming growth factor *β*, and bone morphogenic proteins (BMP-2, 4 and 7). The accumulation of these molecules is thought to induce the initiation of the secondary enamel knots at the sites that mark cusp formation during the tooth development [3, 5].

Although innumerous case reports regarding the prevalence of dens evaginatus arising from the occlusal, lingual, labial, and buccal surfaces exist, reports of a similar description in dental literature detailing protrusion of tooth from the mesial surface were not found [8–12]. Regarding the etiology, we believe that the pathogenesis is similar to the other DE presentations which include both genetic and environmental factors, and this case could have been just a rare oddity. And rightly so, a designation does not exist for such an appearance for which we suggest a term “dental horn.”

## 4. Conclusion

In conclusion, we report a never before described clinical finding of an unilateral proximal horn-like structure of the mandibular right first premolar as a result of an anomalous development of the enamel, and probably the underlying dentine. The purpose of this report was to highlight this unique finding and its probable pathogenesis to increase awareness among the dental fraternity.
